# Trajectories of Self-Rated Health of Chinese Elders: A Piecewise Growth Model Analysis

**DOI:** 10.3389/fpsyg.2019.00583

**Published:** 2019-03-19

**Authors:** Guangming Li, Guiyun Hou, Guohong Xie, Dong Yang, Hu Jian, Weijun Wang

**Affiliations:** ^1^Guangdong Key Laboratory of Mental Health and Cognitive Science, Center for Studies of Psychological Application, School of Psychology, South China Normal University, Guangzhou, China; ^2^School of Public Finance and Public Administration, Jiangxi University of Finance and Economics, Nanchang, China; ^3^Department of Psychology, Clinical and Research Institute on Addictions, University at Buffalo, State University of New York, Buffalo, NY, United States

**Keywords:** self-rated health, piecewise growth model, elderly people, activities of daily living, gender

## Abstract

This study used piecewise growth modeling to describe the developmental trajectories of self-rated health (SRH) in the elderly and longitudinal associations with activities of daily living (ADL), educational level, economic status, age, and gender. Data were drawn from the Chinese Longitudinal Healthy Longevity Survey (CLHLS), collected over 12 years (from 2002 to 2014) at five waves. A total of 16,064 Chinese elders (57.4% females) were analyzed. Results showed two phases of development for SRH; specifically, the decreasing trend of SRH was from slow (in the first phase, waves 1 to 3) to fast (in the second phase, waves 3 to 5). Descriptives showed that the turning point age was at the age of 83.69 (range = 68 to 116, median age = 82 years old). ADL were positively associated with SRH within each time point (wave of data). Female elders had a higher initial state (i.e., worse) of SRH than did male elders, and poorer economic status was associated with worse initial status of SRH.

## Introduction

Self-rated health (SRH) in elderly people has been documented and understood as an important indicator of the elder’s overall health over time (Cullati et al., 2014; Meng and D’Arcy, 2016). In 2016, 10.8% of Chinese population were aged 65 years or older (National Bureau of Statistics of China, 2016). The population of this group of citizens has been growing. The public access to the Chinese Longitudinal Healthy Longevity Survey (CLHLS; Zeng et al., 2017) allowed us to examine the longitudinal trajectories of SRH and associations with several potential, covariate variables that may contribute to a long, healthy life for Chinese elders they deserve. In this study, we examined the longitudinal trajectories of SRH and associations between SRH and elders’ activities of daily living (ADL), educational level, economic conditions, age, and gender in a large Chinese community sample. We trust the transparency of this public data (i.e., CLHLS) and the voices (perceptions) of the elders themselves.

The elderly population is increasing globally (Arifin and Hogervorst, 2015). SRH is a primary indicator of their health (Mossey and Shapiro, 1982; Cullati et al., 2014). SRH often shows sensitivity for preclinical disease which can hardly be captured by the measurement (Mcfadden et al., 2009). SRH typically has a high reliability and validity (Mcfadden et al., 2009; Arifin and Hogervorst, 2015). There is increasing evidence that one’s future physical health, functional limitations, quality of life, morbidity and mortality has been related to SRH (Meng and D’Arcy, 2016). For example, Frankenberg and Jones (2004) found that SRH was a significant predictor of mortality. Compared with the people who reported a good SRH, those who perceived their own health status as poor were more likely to die in the following years. Maharlouei et al. (2016) found that SRH was associated with education level, household monthly income, chronic illness, psychological health disorders and oral disorders in an Iranian sample, which indicated that SRH could be an effective predictor of public health.

Given that SRH has an important effect on public health, it is important to identify the determinants of SRH. These may include gender (Tarleton et al., 2017), education level (Waller et al., 2015), age, socioeconomic status (Cullati et al., 2014), and ADL (Waller et al., 2015; Fonta et al., 2017; Tarleton et al., 2017). However, findings were mixed. For example, some studies suggested gender differences in SRH (Idler, 2003), but some did not (Rohlfsen and Jacobs, 2014). Similarly, most studies have showed positive associations between ADL and SRH (Fonta et al., 2017; Tarleton et al., 2017), nevertheless, some scholars have not found this association. For example, Boyington et al. (2008) have not found an association between SRH and ADL in a sample of Black American stroke survivors. These inconsistencies may have occurred due to the variations of the studied data or other reasons. However, it is possible that different phases of development for SRH may also somewhat explain these inconsistencies over time. This study intended to identify the phases of development for SRH in Chinese elderly and examine the relationship between these variables and SRH over each possible phase of development using piecewise growth modeling.

Few studies have discussed SRH from a longitudinal perspective, rather, most SRH studies have been conducted using cross-sectional data (Waller et al., 2015; Tarleton et al., 2017). Cross-sectional studies cannot reflect the developmental trajectories of SRH and the differences among individuals longitudinally. What’s more, cross-sectional data cannot show whether these factors might have slowed the decreasing rate of SRH over aging. For example in China, Cheng et al. (2015) used a cross-sectional study in a sample of urban Chinese women to investigate the associations between SRH and subjective health complaints (SHC) and health-promoting lifestyles in a sample of urban Chinese women. The authors found that health-promoting lifestyles were related to higher SRH but lower SHC. Another study (Sereny and Gu, 2011) examined the relationship between living arrangement concordance and SRH among institutionalized and community-residing older adults in China, and they found that for both institutionalized and community-residing older adults, living arrangement concordance increased the likelihood of rating SRH as good. In general, SRH has not been a focus in China. No published longitudinal studies have looked into SRH in Chinese cultures, to our knowledge.

In sum, this study intended to examine (1) the trajectory patterns and the rate of change of SRH over time in Chinese elders, and (2) the longitudinal associations between SRH and ADL, educational level, economic status, age, and gender. It is hoped that the present study could provide scientific research evidence for the importance of improving the quality of life in elderly people considering their own perceptions of SRH.

## Materials and Methods

### Participants

Participants included a large, random sample of Chinese elders involved in the CLHLS (Zeng et al., 2017). The CLHLS data were collected at seven waves over 16 years, first in 1998, and then in 2000, 2002, 2005, 2008, 2011, and 2014. The CLHLS examined Chinese elders’ health conditions, everyday functioning, self-perceptions of health status and quality of life, life satisfaction, mental attitude, and feelings about aging (Zeng et al., 2017). At the 1998 wave, elders at 80 years old or older were recruited, but starting from 2002, elders aged 65–79 were also recruited. In this study, we analyzed the most recent five waves data (i.e., 2002, 2005, 2008, 2011, and 2014). (the 2002 wave data was treated as baseline in the present study; newly recruited sample in the following waves were not included in the study). There were 16,064 elders included in this study (57.4% females) aged 65 to 120 (*M* = 86.33, *SD* = 11.70) at baseline. Men averaged 83.76 (*SD* = 10.80) and women 88.23 (*SD* = 11.98) years of age. There were 1,680 elders contributing complete data at all five waves (10.5% of the sample, 881 females and 799 males). 834 had data at four waves (5.2%; 451 females and 383 males), 1,677 had data at three waves (10.4%; 915 females and 762 males), 3,984 had data at two waves (24.8%; 2,235 females and 1,749 males), and 7,889 had data at one wave (49.1%; 4,737 females and 3,152 males). Because our models involved within time point (wave) associations, we included these elders with 1 time point data in our analysis.

Chinese Longitudinal Healthy Longevity Survey is accessible to the public for research purposes (Zeng et al., 2017). Permission to use the data for this study was obtained, and this study was approved by the South China Normal University research ethics board (Institutional Review Board).

### Measures

#### Self-Rated Health

Self-rated health was measured using the sing-item question, “What do you think of your own health?” on 5 point response scales (ranging from 1 = *Very good* through 5 = *Very bad*). Higher scores indicated lower in elders’ SRH.

#### Activities of Daily Living

Activities of daily living was measured using the Katz index (Katz et al., 1963; Fong and Feng, 2016). The Chinese ADL version (since 2002) included 14 items (e.g., “Can you cook alone if you need it?”), rated on 3 point scales (1 = *Yes*; 2 = *A little difficult*; 3 = *Unable to do so*). We created a composite score for each elder by taking the sum of all these 14 items (scores were reversed whenever necessary), with higher scores indicating more help needed. Internal consistency for the ADL items was good at each wave (Cronbach’s alpha = 0.94 at all five waves).

#### Covariates

Covariate variables included gender (0 = *Male*; 1 = *Female*), age, self-reported education level (years of schooling) and economic status (from 1 = *very rich* to 5 = *very poor*), assessed at the 2002 wave (in this study, treated as baseline).

### Analytic Strategy

Analyses were performed in Mplus (Version 8.2) using maximum likelihood estimation with robust standard errors (Muthén and Muthén, 1998/2017). We used two steps. Step 1, we conducted a series of unconditional growth models to examine the trajectory patterns of Chinese elders’ SRH over time. These growth models included linear growth model, quadratic growth model, and piecewise growth model. The time scores for the slope growth factor were coded to reflect years past baseline (0, 3, 6, 9, and 12). Our preliminary analyses showed that a piecewise growth model fit the data best (see below). Model fit was determined based on conventional standards (e.g., Lo et al., 2001; Nylund et al., 2007). We examined the chi-square test of model fit, root mean square error of approximation (RMSEA), CFI/TLI, standardized mean root square residual (SRMR), and Bayesian information criterion (BIC). In the piecewise growth modeling, two phases of development were captured. The first phase of development included the first three waves. The second phase of development included the last three waves. Step 2, we examined predictions of the time-varying covariates ADL within each time point, and of the time-invariant covariates gender, age, educational level, and economic status in the two-stage piecewise growth model.

## Results

### Descriptives

Descriptive statistics and bivariate correlations among primary variables are displayed in Table 1.

**Table 1:** Descriptives and bivariate correlations among primary variables.

Descriptive statistics					Correlations													
Variable	Min	Max	*M*	*SD*	1	2	3	4	5	6	7	8	9	10	11	12	13	14
1. SRH2002	1.00	5.00	2.60	0.92	–													
2. SRH2005	1.00	5.00	2.63	0.95	0.24**	–												
3. SRH2008	1.00	5.00	2.62	0.94	0.18**	0.23**	–											
4. SRH2011	1.00	5.00	2.66	0.98	0.21**	0.20**	0.19**	–										
5. SRH2014	1.00	5.00	2.69	0.94	0.19**	0.22**	0.18**	0.33**	–									
6. ADL2002	14.00	42.00	21.28	7.86	0.29**	0.13**	0.10**	0.08**	0.04	–								
7. ADL2005	14.00	42.00	20.47	7.74	0.12**	0.32**	0.13**	0.10**	0.13**	0.61**	–							
8. ADL2008	14.00	42.00	19.44	7.17	0.10**	0.13**	0.28**	0.13**	0.09**	0.47**	0.59**	–						
9. ADL2011	14.00	42.00	19.86	7.58	0.10**	0.11**	0.15**	0.30**	0.15**	0.38**	0.46**	0.59**	–					
10. ADL2014	14.00	42.00	19.99	7.63	0.12**	0.14**	0.11**	0.18**	0.32**	0.28**	0.38**	0.45**	0.61**	–				
11. Education	0.00	25.00	2.02	3.50	-0.08**	-0.7**	-0.04*	-0.10**	-0.06*	-0.17**	-0.14**	-0.12**	-0.13**	-0.12**	–			
12. Economic	1.00	5.00	3.00	0.68	0.25**	0.14**	0.12**	0.09**	0.12**	0.08**	0.04**	0.02	0.02	0.02	-0.17**	–		
status
13. Age	65.00	120.00	86.33	11.70	0.05**	0.04**	0.05**	0.00	-0.02	0.61**	0.57**	0.55**	0.46**	0.39**	-0.23**	0.03**	–	
14. Gender	0.00	1.00	–	–	0.06**	0.06**	0.04**	0.08**	0.04	0.23**	0.18**	0.15**	0.13**	0.14**	-0.40**	0.04**	0.19**	–


*^∗^Correlation is significant at the 0.05 level (2-tailed). ^∗∗^Correlation is significant at the 0.01 level (2-tailed). SRH, self-rated health; ADL, activities of daily living*.

### Unconditional Growth Models

**FIGURE 1:**
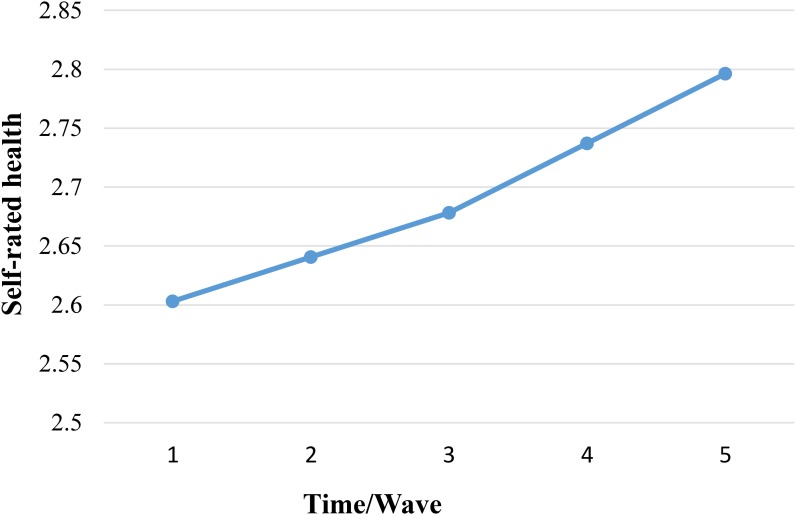
Two phases of development in self-rated health, unconditional piecewise model. Higher scores indicated lower in Chinese elders’ SRH condition.

We examined the model fit indicators for the unconditional growth models (i.e., linear, quadratic, and piecewise). Results showed that the piecewise growth model fit the data best (Table 2). Two phases of development for SRH were identified (Table 3 and Figure 1). The average starting amount of SRH (the intercept) was 2.60 units, *b* = 2.603 (0.007), *p* < 0.001, and the average growth in Chinese elders’ SRH was 0.01 in the first phase (i.e., the slop 1; 2002 wave through 2008 wave), *b* = 0.013 (0.002), *p* < 0.001, and the average growth in Chinese elders’ SRH was 0.02 in the second phase (i.e., the slop 2; 2008 wave through 2014 wave), *b* = 0.020 (0.004), *p* < 0.001. As expected, Chinese elders’ SRH decreased by 0.01 units each studied wave period in the first phase and decreased by 0.02 units in the second phase each studied wave period. The rate of change (declining) of SRH was from slow to fast. Results also indicated that there was non-trivial variation in the amount of Chinese elders’ SRH at the initial time point, *b* = 0.256 (0.022), *p* < 0.001, and Chinese elders’ SRH may be varied over time both in the first phase, *b* = 0.003 (0.001), *p* = 0.022, and in the second phase, *b* = 0.009 (0.002), *p* < 0.001. The amount of Chinese elders’ SRH at the initial time of measurement was associated with changes over time in the first phase (but not with changes in the second phase). In this study, the turning point of development for SRH was at time point 3 (the 2008 wave). The average age of the study elders was 83.69 (range = 68 to 116, the median age was 82 years old).

**Table 2:** Model fit indicators for linear, quadratic, and piecewise growth models.

Model	*χ*^2^ (df)	*P*-value	CFI/TLI	RMSEA (90% CI)	SRMR	BIC
Linear	32.586 (10)	0.0003	0.980/0.980	0.012 (0.008–0.017)	0.026	80616.746
Quadratic	18.622 (6)	0.0049	0.989/0.982	0.012 (0.006–0.018)	0.018	80641.230
Piecewise	8.684 (6)	0.1921	0.998/0.996	0.005 (0.000–0.013)	0.010	80631.293


**Table 3:** Unconditional model results of the piecewise growth analysis model.

	Estimate (S.E.)	95% CI
S1 with I	-0.013 (0.005)^∗^	[-0.023, -0.003]
S2 with I	0.003 (0.004)	[-0.005, 0.011]
S2 with S1	-0.002 (0.001)	[-0.004, 0.001]
Means		
I	2.603 (0.007)^∗∗∗^	[2.589, 2.618]
S1	0.013 (0.002)^∗∗∗^	[0.008, 0.017]
S2	0.020 (0.004)^∗∗∗^	[0.011, 0.028]
Variances		
I	0.256 (0.022)^∗∗∗^	[0.212, 0.300]
S1	0.003 (0.001)^∗^	[0.000, 0.006]
S2	0.009 (0.002)^∗∗∗^	[0.006, 0.013]


*S, slope; I, intercept. ^∗∗∗^*p* < 0.001; ^∗∗^*p* < 0.01; ^∗^*p* < 0.05*.

### Conditional Piecewise Growth Model

**FIGURE 2:**
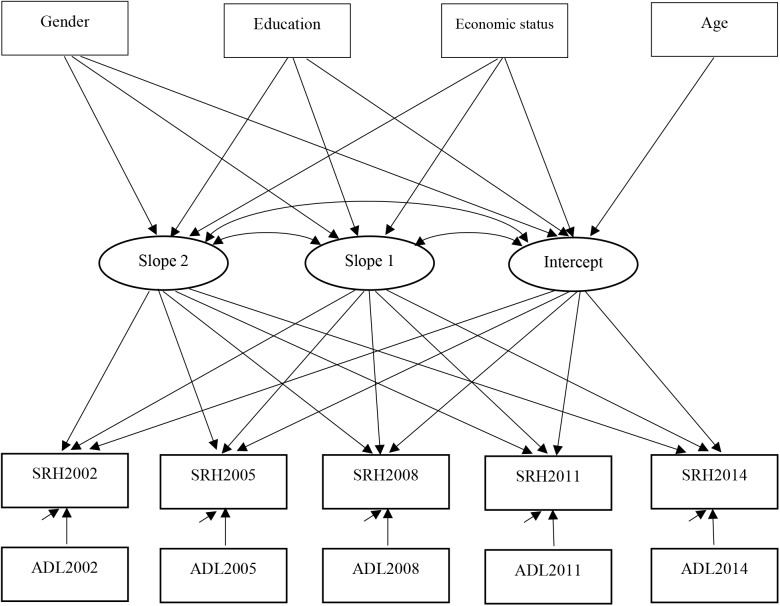
The conceptual conditional piecewise growth analysis model.

We tested the effects of ADL (time varying) within each wave (within person) and also the time-invariant covariates gender, age, education level, and economic status (i.e., between person; predicting the intercept and the two slope factors; age only predicted intercept) in our two-stage piecewise growth model. Figure 2 provides a conceptual conditional piecewise growth model. Results are displayed in Table 4. ADL was positively associated with SRH within each time point. Gender predicted the intercept but not the slopes. Female elders reported a higher (i.e., worse) initial mean score of SRH than male elders. Economic status predicted the intercept and the slope 1 (but not slope 2). Elders who reported a poorer economic status also reported a higher (i.e., worse) initial status of SRH and sharper decrease in perceived health in the first phase than elders who reported a better economic status. As expected, age was negatively associated with the intercept of SRH. Chinese elders at an older age rated their health status worse. Education did not predict the initial status or the change of elders’ SRH. The inter-individual differences in the intercept and the slope 2 remained, but no variability emerged for slope 1, and we did not display. There were no significant correlations between the slope 1, slope 2, and the intercept.

**Table 4:** Conditional model results of the piecewise growth analysis model.

	Estimate (S.E.)	95% CI
S1 with I	-0.002 (0.007)	[-0.017, 0.012]
S2 with I	-0.003 (0.004)	[-0.010, 0.005]
S2 with S1	-0.001 (0.001)	[-0.003, 0.002]
Covariates predicting the intercept		
Age	-0.013 (0.002)***	[-0.017, -0.009]
Education	-0.005 (0.006)	[-0.017, 0.007]
Gender	0.123 (0.044)**	[0.036, 0.210]
Economic status	0.256 (0.032)***	[0.193, 0.319]
Covariates predicting the slope 1		
Education	0.001 (0.001)	[-0.002, 0.004]
Gender	-0.011 (0.010)	[-0.031, 0.010]
Economic status	-0.017 (0.008)*	[-0.032, -0.002]
Covariates predicting the slope 2		
Education	-0.002 (0.001)	[-0.005, 0.001]
Gender	-0.015 (0.011)	[-0.036, 0.006]
Economic status	0.003 (0.008)	[-0.012, 0.018]
Time-varying covariates		
SRH2002 < –ADL2002	0.061 (0.007)***	[0.047, 0.075]
SRH2005 < –ADL2005	0.062 (0.004)***	[0.054, 0.070]
SRH2008 < –ADL2008	0.060 (0.005)***	[0.051, 0.070]
SRH2011 < –ADL2011	0.052 (0.003)***	[0.046, 0.057]
SRH2014 < –ADL2014	0.043 (0.003)***	[0.037, 0.049]


*SRH, self-rated health; ADL, activities of daily living; S, slope; I, intercept. ^∗∗∗^*p* < 0.001; ^∗∗^*p* < 0.01; ^∗^*p* < 0.05*.

To ensure that the pattern of results was robust, we tested the piecewise growth model using the sample who had five wave data (*n* = 1,680). Similarly, two phases of development for SRH was identified. Specifically, the average starting amount of SRH (the intercept) was 2.39 units, *b* = 2.388 (0.020), *p* < 0.001, and the average growth in elders’ SRH was 0.02 in the first phase (i.e., the slop 1; 2002 wave through 2008 wave), *b* = 0.017 (0.004), *p* = 0.002, and the average growth in elders’ SRH was 0.03 in the second phase (i.e., the slop 2; 2008 wave through 2014 wave), *b* = 0.033 (0.005), *p* < 0.001. Results also indicated that there was variation in the amount of Chinese elders’ SRH at the initial time point, *b* = 0.178 (0.037), *p* < 0.001, and elders’ SRH varied over time in the second phase, *b* = 0.008 (0.002), *p* < 0.001, but not in the first phase (*p* = 0.498). Results provide the first evidence for two phases of development for SRH in Chinese elderly and help to understand their perceptions of their own health functioning.

We did not provide a hypothesis for the age-specific relationship between SRH and ADL of the Chinese elderly sample. However, we conducted a series of linear growth models using the data widetolong command to examine the possible age-specific relationship (due to the limited portion of complete data over the five waves, we were unable to examine the age-specific relationship using piecewise models). To do so, we categorized the sample into four age groups – young elders (aged 65–79; *n* = 4,889), octogenarians (aged 80–89; *n* = 4,239), nonagenarians (aged 90–99; *n* = 3,747), and centenarians (aged 100–120; *n* = 3,189). Results showed that there were positive associations between SRH and ADL for each of these four groups of Chinese elderly sample. However, the estimated relationship weakened with age (*b*s = 0.063 [0.002], 0.053 [0.002], 0.044 [0.002], and 0.043 [0.002], respectively; all *p’*s < 0.001).

## Discussion

This study used piecewise growth modeling to examine the developmental trajectories of Chinese elders’ SRH and the effect of several key covariates, including ADL, gender, age, educational level, and economic status. Our piecewise growth analysis models showed that the Chinese elderly people perceived their health decreasing over time (12 years in the present study, from 2002 through 2014). This finding is consistent with what the literature has documented in other cultures (e.g., Cullati et al., 2014; Hanibuchi et al., 2016). The decreasing trend could be explained by a biological, aging process (Fonta et al., 2017). However, the present study showed that the decreasing trend of Chinese elders’ SRH was non-linear. Specifically, piecewise growth modeling revealed two phases of development (decreases) of SRH: first declined slowly, and then declined rapidly. The declining trend of SRH might be consistent with trend of cognition development in the elderly. Petersen et al. (2001) suggested that an extreme form of cognitive impairment is Alzheimer’s disease, and mild cognitive impairment represents early-stage Alzheimer disease, meaning that a mild cognitive impairment is between normal cognitive and Alzheimer’s disease, once the stage of mild cognitive impairment is exceeded, the cognitive function declines rapidly, which seriously affects the function of elders’ daily life. It is possible that, like the cognitive function, before the rapid decline in SRH, there is a stage of mild decline (in SRH). In our sample, the turning point of development of SRH occurred at an average age of 83.69 years old (range = 68 to 116, the median age was 82 years old). Any prevention and intervention efforts to address health in the elderly should take measures to slow the decline of SRH before this rapid decline starts to occur.

This study provides evidence that several variables contribute to Chinese elders’ SRH. Consistent with other studies (e.g., Fonta et al., 2017), we found that ADL was positively associated with SRH. Low levels of physical activities can lead to multiple organ system failure and increase stress (Tarleton et al., 2017). Daily help and care should be available for the elderly people; however, they also should be encouraged to promote their effort to be involved in activities of their everyday living (including their personal issues) as much as possible if they are able to.

This study showed that Chinese elderly people with a poorer economic status had a worse initial SRH (Gu et al., 2017). Several other SRH studies have considered socioeconomic status as a protector factor of SRH (Cullati et al., 2014). Lower socioeconomic status is often associated with poorer physical and psychological health (Lindström et al., 2017). The elderly with high socioeconomic status often have broader access to serviceable resources. People from low socioeconomic backgrounds (and areas) are less like to seek health care (Lindström et al., 2017). It is notable that in this study a poor economic status also predicted a steeper change (worse) in the first phase of SRH. Special attention should be placed to the elderly people with low socioeconomic status, and assistance and help should be in place for them.

This study also showed that female elders had a worse initial state of SRH than did male elders in the sample. Rohlfsen and Jacobs (2014) found that working part-time, income, education, and wealth contributed to gender differences in SRH (at baseline). In Rohlfsen and Jacobs’ study, males were more likely than females to have higher income, be wealthy, and have more years of school education, and all these variables were positively related to SRH; whereas females were more likely to not be employed. These findings indicated that gender should be considered in examining SRH, and women’s physical health should be an important focus.

### Limitations

First, a relatively low proportion of the sample (10.5%) contributed complete data at all five waves. We considered several covariate variables (ADL, gender, age, education level, and economic status). Other variables could also be included, for example, family and community information norms that might be related to elders’ SRH. Second, data were elders self-reported and self-evaluated. Future study should include objective measures to assess SRH. Third, in this study, we used piecewise growth modeling. When we entered several covariates, individual differences existed. Future study could use piecewise growth mixture modeling to classify participants into different categories (membership) and then explore the study relationships. The current data did not allow us to do so due to the limited portion of complete data over the five waves. Finally, it seemed sufficient to use linear growth modeling to examine the relationship between SRH and ADL in the current data, but piecewise growth modeling indicated better model fit (Table 2). However, these piecewise growth modeling analyses were data-driven, and our identification of the wave 3 as the turning point between the two phases of development for SRH should be interpreted with caution. We did not provide an explanation for why the wave 3 was the turning point in the real life condition. However, an implication of our analyses is that, any prevention and intervention efforts to address health in the elderly should take measures to slow the decline of SRH before a rapid decline starts to occur.

## Conclusion

In this study, we examined the developmental trajectories of SRH in Chinese elderly people and the predictive role of ADL, gender, age, education level (years of schooling), and economic status. Two phases of development for SRH were identified. The decreasing trend of SRH was slow in the first phase (waves 1 to 3) and was fast in the second phase (waves 3 to 5). Our results suggest that the trend of SRH (declining) should not be understood as linear. ADL was a protective factor for SRH within each time point. Women’s physical health should be an important focus.

## Author Contributions

All authors have contributed significantly to the work and agreed to the current version of the manuscript.

## Conflict of Interest Statement

The authors declare that the research was conducted in the absence of any commercial or financial relationships that could be construed as a potential conflict of interest.
